# Concordance Between Anthropometric Formula Predictions and Chest Radiograph-Confirmed Endotracheal Tube Depth in Young Infants: A Retrospective Cohort Study from Saudi Arabia

**DOI:** 10.3390/jcm15124554

**Published:** 2026-06-12

**Authors:** Volodymyr Mavrych, Kashif Majeed, Saleh Alshehri, Uzma Yasmin, Muhammad Rayyan Kashif, Ayesha Kashif, Warda Mahdi, Raghd Talha, Olena Bolgova

**Affiliations:** 1 College of Medicine, Alfaisal University, Riyadh 11533, Saudi Arabia; 2 Pediatric Intensive Care Unit, King Saud Medical City, Riyadh 12746, Saudi Arabia; 3 Pediatric Emergency, King Saud University, Riyadh 11451, Saudi Arabia

**Keywords:** endotracheal tube, pediatric intubation, insertion depth, concordance correlation coefficient, intensive care

## Abstract

**Background**: Accurate endotracheal tube (ETT) insertion depth is critical in infants and young children, where tracheal malposition carries significant risk. Formula-based depth estimation is widely used at the bedside, but the performance of published formulas in children under two years of age admitted to a general PICU remains poorly characterized. **Methods**: A retrospective, single-center study was conducted at the PICU of King Saud Medical City, Riyadh. A total of 115 patients aged 1–24 months requiring orotracheal intubation were included. ETT depth was predicted using five established formulas: height-based [(H/10)+5], weight-based [W+6], ETT size-based [ETT×3], Lee weight-based [5.5+0.5W], and Lee height-based [3+0.1H]. Agreement between predicted and radiographically confirmed insertion depth was assessed using Lin’s concordance correlation coefficient (CCC), Bland–Altman analysis, and clinical classification of predictions. **Results**: None of the five formulas achieved acceptable concordance (CCC < 0.75 for all). The height-based formula performed best among published formulas, with negligible bias and the highest proportion of clinically acceptable predictions. Both Lee formulas showed near-universal systematic underestimation and are not suitable for this age group. Over half of all intubations resulted in non-ideal ETT position on the first post-intubation chest X-ray. Novel cohort-derived regression equations outperformed all published formulas, with the weight-based equation (Depth = 0.385 × Weight + 9.145) emerging as the strongest predictor of insertion depth. **Conclusions**: No published formula achieved reliable concordance with radiographic ETT depth in children aged 1–24 months. The cohort-derived weight-based formula represents a more accurate bedside tool for this population and warrants prospective external validation. Post-intubation radiographic verification remains essential.

## 1. Introduction

Endotracheal intubation is a fundamental life-saving intervention in pediatric intensive care units (PICUs), yet accurately determining endotracheal tube (ETT) insertion depth remains a critical, unresolved challenge. Inappropriate ETT positioning is associated with significant complications, including inadvertent endobronchial intubation, atelectasis, hypoxia, pneumothorax, and unplanned extubation, all of which may contribute to increased morbidity and prolonged intensive care stay [1,2]. This challenge is particularly pronounced in infants and young children, in whom the trachea is short, anatomically variable, and highly susceptible to displacement even with minimal head and neck movement [3,4].

To facilitate rapid bedside decision-making, several formula-based methods have been developed to estimate optimal ETT insertion depth. Commonly used approaches include the internal diameter-based formula (ETT size × 3), weight-based formulas, and height-based equations such as (height/10) + 5 cm [5,6,7]. Despite their widespread adoption, these formulas are largely derived from limited datasets and specific populations, raising concerns regarding their generalizability and accuracy across diverse pediatric cohorts [6,8,9]. Emerging evidence indicates that formula-predicted ETT depths show variable, often suboptimal concordance with radiographically confirmed ideal positioning, suggesting that reliance on these methods alone may be inadequate to ensure patient safety [1,9].

Radiographic confirmation using chest X-ray remains the gold standard for verifying ETT placement, with the ideal tip location defined as 0.2–2 cm above the carina, typically corresponding to the level of the first to second thoracic vertebrae (T1–T2) in a neutral head position [3,10]. However, routine reliance on radiography presents inherent limitations, including radiation exposure, delays in confirmation, and logistical constraints in emergent or resource-limited settings. Furthermore, dynamic factors such as neck flexion and extension can result in clinically significant ETT tip displacement, often by 1–2 cm, thereby complicating accurate assessment of positioning [3,4]. While adjunctive modalities such as capnography and point-of-care ultrasound are valuable for confirming tracheal placement, they do not reliably determine optimal insertion depth [11].

The British Journal of Anesthesia, in its coverage of airway management in neonates and infants, explicitly endorses Point-of-Care Ultrasound (POCUS) as a useful bedside adjunct for confirming tracheal intubation [12]. Systematic evidence now supports its role in confirming tracheal ETT placement across a broad range of pediatric populations: a comprehensive systematic review of 33 studies encompassing 1934 ultrasound examinations in neonates, infants, and older children reported a diagnostic accuracy of 90.6–100% for confirming successful endotracheal intubation [13]. In neonates specifically, where the trachea is shortest and malposition most consequential, a dedicated meta-analysis demonstrated that POCUS can reliably confirm ETT placement while substantially reducing radiation exposure and confirmation delays compared with chest radiography [14]. Prospective validation in the pediatric ICU setting has similarly demonstrated ultrasound accuracy exceeding 97% for confirming correct ETT placement against a chest radiographic reference standard, with high inter-rater reliability [15]. However, a critical and often underappreciated limitation is that ultrasound confirmation of tracheal placement does not confirm optimal insertion depth. Airway ultrasound’s accuracy in quantifying ETT tip-to-carina distance varies widely (66.7–100%) [13], and, from a neonatologist’s perspective, reliable tip localization by ultrasound alone remains insufficient to determine whether the tube is positioned at a safe distance above the carina in clinical practice [16]. Accordingly, while POCUS offers a rapid, radiation-free method for confirming that the tube is in the trachea rather than the esophagus—a particularly valuable capability in the emergent and resource-limited settings characteristic of many PICUs—it does not replace chest radiographic verification of insertion depth, and the fundamental challenge of determining optimal ETT depth at the bedside remains unresolved.

Artificial intelligence (AI) and machine learning (ML) approaches represent a rapidly evolving frontier in addressing the limitations of both formula-based depth prediction and manual radiographic interpretation. On the radiographic side, deep learning models trained on large chest radiograph datasets have demonstrated the ability to automatically identify and localize the ETT tip relative to the carina with accuracy comparable to that of radiologists: a landmark study training a convolutional neural network on nearly 23,000 ICU chest radiographs achieved sensitivity and specificity exceeding 90% for detecting critically low ETT positions, and predicted ETT-to-carina distance within 1 cm in most cases [17,18]. Such automated radiographic assessment tools have clear potential as real-time clinical decision-support systems, capable of flagging ETT malposition on post-intubation chest radiographs without the delays inherent to formal radiology reporting—a capability particularly relevant in the high-acuity, time-critical environment of the pediatric ICU. On the depth-prediction side, ML models integrating multiple demographic and anthropometric variables have consistently outperformed conventional single-parameter formulas. In pediatric patients under seven years of age, ML approaches—including random forest, elastic net, support vector machines, and artificial neural networks—achieved optimal ETT depth prediction in up to 79% of cases, compared with only 44.4% for the height-based formula and 58.5% for the tube-ID formula using the same dataset [19]. A subsequent study applying ML specifically to predict the risk of inappropriate ETT depth in pediatric patients under seven years demonstrated that ML models significantly outperformed all formula-based comparators, including age-based, height-based, and tube internal diameter methods [20]. Collectively, these findings suggest that the ceiling of linear formula-based depth estimation has been reached, and that ML-driven, multivariate, and potentially non-linear prediction frameworks represent the most promising direction for improving individual-level accuracy in ETT depth estimation—particularly in the youngest and most anatomically variable patients, where formula-based approaches have proven least reliable [17,19,20].

Recent advances in predictive modeling, including the application of machine learning techniques, have demonstrated improved accuracy in estimating ETT size and depth by integrating multiple demographic and anthropometric variables [17]. Similarly, imaging-based studies utilizing computed tomography have proposed refined formulas that outperform traditional single-parameter methods [7]. Nonetheless, these approaches require external validation and may lack practicality for routine bedside implementation. Importantly, anthropometric characteristics and airway dimensions vary across populations, potentially influencing the performance of existing predictive models [6]. Despite this, there is a notable absence of studies evaluating the accuracy and concordance of ETT depth prediction formulas within the Saudi pediatric population. Given regional differences in growth patterns and body proportions, extrapolating data from other populations may lead to systematic inaccuracies in clinical practice [2,8,9]. Prior investigations suggest that incorporating multiple anthropometric parameters (such as weight, height, and age) may enhance predictive accuracy compared with traditional formulas [21,22], supporting the need for population-specific model development.

Therefore, this study aims to (1) evaluate the concordance between five established formula-predicted ETT insertion depths and radiographically confirmed ideal positioning in Saudi pediatric patients aged 1–24 months; (2) derive cohort-specific regression equations as a population-calibrated alternative to published formulas; and (3) examine the variation in predictor–depth relationships across age strata within this population. Establishing a more reliable and clinically applicable method for determining ETT insertion depth could reduce airway-related complications and optimize airway management practices in pediatric critical care settings [10].

## 2. Materials and Methods

### 2.1. Study Design and Setting

This was a retrospective, observational, cross-sectional study conducted at the Pediatric Intensive Care Unit (PICU) of King Saud Medical City (KSMC), Riyadh, Saudi Arabia. Data were collected from electronic medical records spanning January 2024 to December 2025, the PICU database, and the Picture Archiving and Communication System (PACS).

### 2.2. Study Population

The study population comprised pediatric patients aged 1 month to 2 years who were admitted to the PICU and required mechanical ventilation via endotracheal intubation. Of 1562 total admissions, 528 were ventilated, and 216 were under 2 years of age. After applying exclusion criteria—including 73 patients intubated outside the hospital or in the emergency room, with tube adjustments made before admission to the PICU; 15 with poor-quality X-rays; and 13 with significantly rotated or flexed films, agreed upon by both reviewers—a final cohort of 115 patients was included in the analysis.

### 2.3. Inclusion Criteria

Patients were eligible if they were between 1 month and 2 years of age at the time of intubation, were intubated with an oral endotracheal tube, had a chest X-ray performed within 4 h of intubation, had documented anthropometric data (weight and height/length) recorded at the time of admission to the hospital, had documented ETT internal diameter and insertion depth at the lip level, and had no documented ETT repositioning between intubation and the first chest X-ray.

### 2.4. Exclusion Criteria

Patients were excluded if they were neonates (<28 days of age), had known congenital airway anomalies, had a tracheostomy or prior airway surgery, or had a chest X-ray of inadequate quality (carina not clearly visible, ETT tip obscured, or significant rotation of the chest). Additional exclusion criteria included missing critical data (weight, height, ETT size, or insertion depth) or a chest X-ray performed with the neck in extreme flexion, extension, or rotation as documented in the radiology report.

### 2.5. Data Collection

Demographic and clinical data included patient age at intubation (in months), sex, indication for intubation (respiratory failure, neurological cause, post-operative, shock/sepsis, or other), intubation urgency (elective vs. emergent), and number of intubation attempts. Anthropometric data, weight (kg) and height/length (cm), were obtained from the most recent measurement recorded within 48 h of intubation. ETT data collected included internal diameter (mm) and insertion depth at the lips. All tubes in this cohort were uncuffed, consistent with standard practice for this age group [23].

Additional clinical data collected included whether ETT repositioning was required following the X-ray and whether reintubation was required within 48 h. Complications recorded during the admission included endobronchial intubation, hypoxia or desaturation, pneumothorax, atelectasis, peritubular ETT leak, and other events.

### 2.6. Formula-Based Depth Predictions

For each patient, the predicted ETT insertion depth was calculated using five established formulas:

- Height-based: (Height in cm ÷ 10) + 5. This formula was derived from radiographic and bronchoscopic airway measurements in children, in which the distance from the teeth to the mid-trachea was found to correlate strongly with height, leading to its recommendation for clinical use [6]. The original derivation cohort comprised children aged 3–16 years; its applicability to infants under 2 years has therefore been specifically examined in this study [1].
- Weight-based: Weight in kg + 6, also described as the ‘weight+6’ rule, one of the most widely used bedside formulas for ETT depth estimation in the pediatric literature [1,5,12].
- ETT size-based: ETT internal diameter (mm) × 3. A prospective evaluation of this rule in a PICU population found that 15–25% of tubes were malpositioned, leading to the conclusion that a more reliable method is necessary [5].
- Lee weight-based: 5.5 + (0.5 × body weight in kg). Derived from regression analysis of sagittal neck CT images in 1111 children; designed for use in children younger than 1 year [7].
- Lee height-based: 3 + (0.1 × height in cm). Derived from the same CT-based dataset, designed for use in children older than 1 year [7].

Three of the five formulas (height-based, weight-based, and ETT size-based) have been previously benchmarked against radiographic ETT position in children aged 29 days to 2 years [1]. The two Lee formulas are included as additional comparators in the present study. All calculations were performed using a standardized electronic spreadsheet with data validation to minimize computational errors.

### 2.7. Radiographic Assessment

Radiographically confirmed ETT tip position was determined by measuring the distance from the ETT tip to the carina along the tracheal axis on anteroposterior (AP) or posteroanterior (PA) chest X-rays, using calibrated PACS measurement tools. The carina was identified as the point of bifurcation of the main bronchi, and all measurements were recorded to the nearest 0.1 cm. Chest radiography is the established gold standard for confirming ETT placement in children [1,5,12].

Each radiograph was independently assessed by two reviewers (a pediatric radiologist and a trained PICU physician), blinded to formula-predicted depths, clinical outcomes, and each other’s measurements. Inter-rater reliability was assessed using the intraclass correlation coefficient (ICC). Measurements were performed in separate sessions with no cross-reviewer communication prior to data entry, and the ICC (2,1) model (two-way mixed-effects, absolute agreement, single measures) was used as the most conservative estimate for two fixed reviewers. The high ICC is consistent with the strict inclusion criteria applied in this study: only radiographs with an unobscured carina, a clearly visible ETT tip, and a neutral head position were retained, and 28 films were excluded for inadequate image quality or positioning. Under these conditions, the tip-to-carina measurement is geometrically straightforward. Discrepancies greater than 0.2 cm between reviewers were resolved by joint consensus measurement; if consensus was not reached, a third independent reviewer provided a measurement, and the average of the two closest values was used. ETT tip position was categorized as ideal (0.5–2.0 cm above the carina), too deep (<0.5 cm above the carina or endobronchial), or too shallow (>2.0 cm above the carina), consistent with definitions used in the literature [1,5].

### 2.8. Outcome Measures

The primary outcome was Lin’s concordance correlation coefficient (CCC) between the height-based formula prediction [(height in cm/10) + 5] and the radiographically confirmed ETT insertion depth at the lip level. The CCC, originally described by Lin [24], quantifies both precision (the Pearson correlation component) and accuracy (how closely predictions lie on the line of perfect agreement) on a scale from −1 to 1. Values > 0.90 indicate good concordance, 0.75–0.90 moderate concordance, and <0.75 poor concordance.

Secondary outcomes included Lin’s CCC for each of the remaining four formulas (weight-based, ETT size-based, Lee weight-based, and Lee height-based); the proportion of clinically acceptable formula predictions, defined by the absolute difference between each formula’s predicted depth and the radiographically confirmed insertion depth for that patient (≤0.5 cm as ideal, >0.5 to ≤1.0 cm as acceptable, and >1.0 cm as unacceptable); Pearson correlation coefficients; mean absolute error (MAE); root mean square error (RMSE); Bland–Altman analysis for bias and 95% limits of agreement; and the frequency and direction of predicted ETT malposition (too deep or too shallow). The rate of ETT repositioning required after the post-intubation X-ray and complications related to ETT position were also recorded.

### 2.9. Statistical Analysis

Data analysis was performed using R (version 4.5.3). Continuous variables are reported as mean ± standard deviation (SD), with median and interquartile range (IQR) where data were not normally distributed; categorical variables are presented as frequencies and percentages. Normality of continuous variables was assessed using the Shapiro–Wilk test.

For each of the five formulas, agreement between formula-predicted and radiographically confirmed ETT insertion depth was assessed using multiple complementary methods: Lin’s concordance correlation coefficient (CCC) with 95% bootstrap confidence intervals (5000 paired resamples) [24], Pearson correlation coefficient, Bland–Altman analysis of systematic bias and 95% limits of agreement (mean difference ± 1.96 SD) [25], mean absolute error (MAE), and root mean square error (RMSE). CCC values > 0.90 were considered good concordance, 0.75–0.90 moderate, and <0.75 poor.

For each formula, predictions were further classified as ideal (absolute error ≤ 0.5 cm), acceptable (>0.5 to ≤1.0 cm), or unacceptable (>1.0 cm) relative to the radiographically confirmed depth, and the direction of predicted malposition (too deep, too shallow, or correct) was tabulated. Inter-rater reliability for radiographic tip-to-carina measurements was assessed using the intraclass correlation coefficient (ICC). Additionally, simple linear regression was performed to derive cohort-specific weight- and height-based equations to predict ETT insertion depth, with radiographically confirmed depth as the dependent variable and weight (kg) or height (cm) as the independent variable. The coefficient of determination (R^2^) and *p*-value were reported for each model. A *p*-value < 0.05 was considered statistically significant for all analyses.

### 2.10. Sample Size

Based on a previously reported ideal insertion rate of 43.3% for the height formula [1], an approximate rate of 40% was assumed. Using a single-proportion formula with 95% confidence and a precision of ±10%, a minimum of 92 patients was required. Accounting for approximately 15% exclusions due to poor X-ray quality or missing data, a target of 110 patients was set. The final cohort of 115 patients exceeded this target.

## 3. Results

### 3.1. Patient Characteristics

Of 1562 total PICU admissions, 528 were ventilated, and 216 were under 2 years of age. Following the exclusion criteria, 115 patients were included in the final analysis. The cohort comprised infants and young children aged 1–24 months with a mean age of 8.5 ± 7.0 months (median 7.0, IQR 2.0–12.0). The majority were male (82 patients, 71.3%), with 33 females (28.7%). Mean weight was 6.45 ± 2.92 kg, mean height/length 65.4 ± 11.7 cm, and mean ETT internal diameter 4.05 ± 0.52 mm. All tubes were uncuffed. The mean X-ray-confirmed ETT insertion depth was 11.63 ± 1.51 cm, and the mean consensus ETT tip-to-carina distance was 0.78 ± 1.04 cm.

Intubation was performed emergently in 97 patients (84.3%) and electively in 18 (15.7%). The most common indication was respiratory failure in 53 patients (46.1%), followed by neurological indications in 23 (20.0%), shock or sepsis in 19 (16.5%), other indications in 11 (9.6%), and post-operative management in 9 (7.8%). First-attempt intubation was successful in 96 patients (83.5%), with 17 (14.8%) requiring a second attempt and 2 (1.7%) requiring 3 or more attempts.

### 3.2. Inter-Rater Reliability

**Figure 1:**
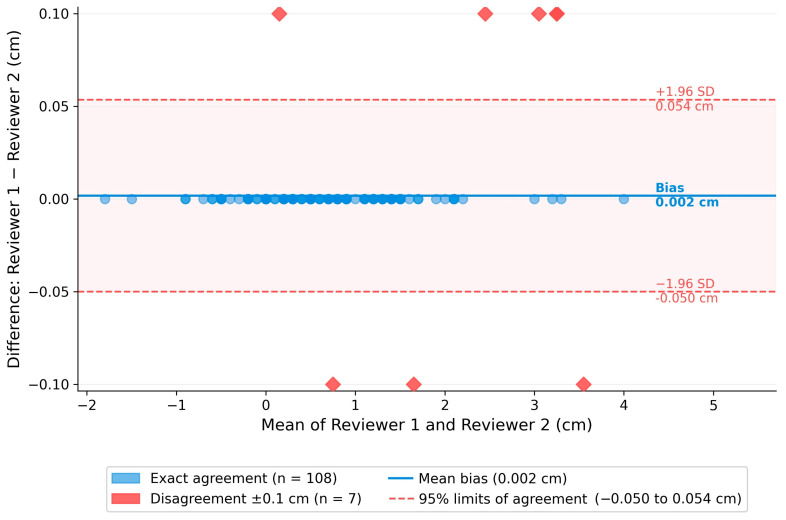
Inter-rater agreement for ETT tip-to-carina distance measurement: scatter plot of Reviewer 1 vs. Reviewer 2 measurements (n = 115). Blue circles represent cases with identical measurements between the two reviewers (n = 108, 94%). Red diamonds represent cases with a disagreement of ±0.1 cm (n = 7, 6%). The solid blue line indicates the mean bias; dashed red lines indicate the 95% limits of agreement.

Agreement between the two independent reviewers for radiographic measurement of the ETT tip-to-carina distance was excellent across all 115 patients. The mean measurements were 0.782 cm (Reviewer 1) and 0.780 cm (Reviewer 2), with a mean difference of 0.002 cm and a standard deviation of differences of 0.026 cm. No case exceeded the pre-specified 0.2 cm threshold for consensus review, and the maximum absolute disagreement in any single case was 0.1 cm. The ICC(2,1) was 0.9997, and Pearson’s r was 0.9997 (Figure 1).

### 3.3. Actual ETT Tip Position on X-Ray

**Figure 2:**
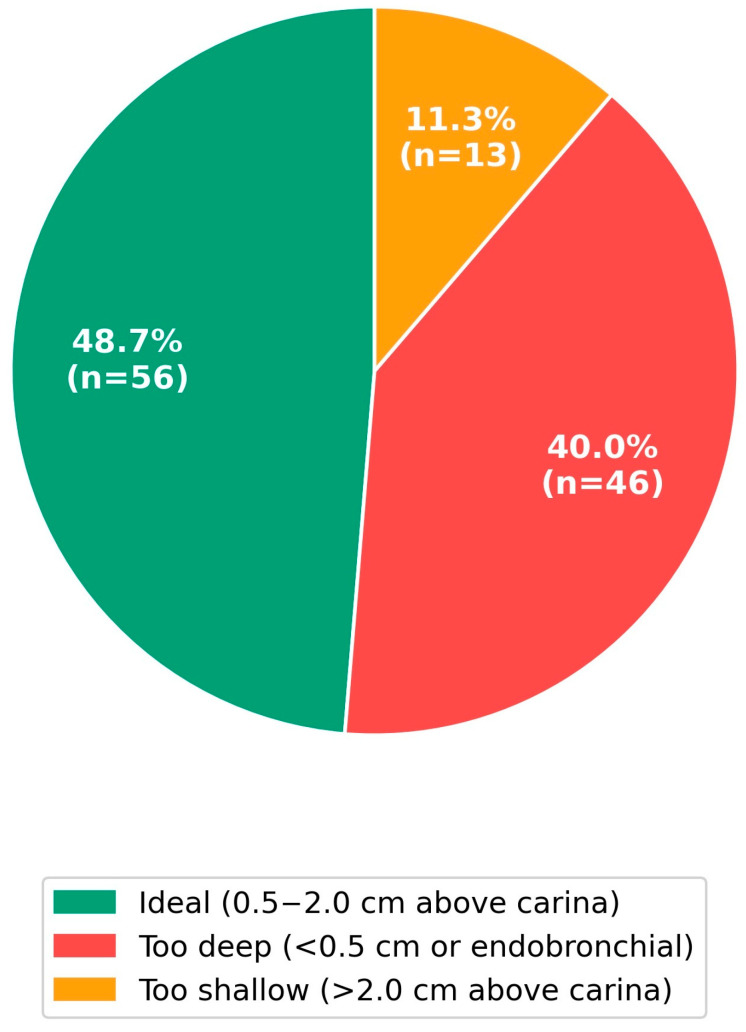
Radiographic ETT tip position at the time of the first post-intubation chest X-ray (n = 115). The ETT tip position was classified based on the final consensus tip-to-carina distance, independent of any formula prediction.

Before evaluating the formulas, the actual radiographic ETT tip position of all 115 patients (classified by the tip-to-carina distance independent of any formula) was as follows: 56 patients (48.7%) were in ideal position (0.5–2.0 cm above the carina), 46 (40.0%) were too deep (<0.5 cm above the carina or endobronchial), and 13 (11.3%) were too shallow (>2.0 cm above the carina) (Figure 2). Thus, 51.3% of intubations were in a non-ideal radiographic position at the time of the first post-intubation X-ray.

### 3.4. ETT Repositioning, Reintubation, and Complications

ETT repositioning was required in 69 patients (60.0%) following review of the post-intubation chest X-ray, while 46 (40.0%) required no adjustment. Reintubation within 48 h was necessary in 5 patients (4.3%). At least one complication was recorded in 95 patients (82.6%), while 20 patients (17.4%) experienced no complications. Note that complications were not mutually exclusive, and individual patients could have more than one. It should be noted that these complications reflect all clinically significant events recorded during the PICU admission and are not attributable solely to ETT malposition. Hypoxia or desaturation and atelectasis, the two most frequent complications, are well-recognized sequelae of critical illness in infants independent of ETT position and cannot be causally attributed to tube malposition in this retrospective design. Complications more directly referable to ETT position include endobronchial intubation (12.2%) and pneumothorax (1.7%), occurring together in 16 patients (13.9% of the cohort).

### 3.5. CCC for All Five Formulas

The primary outcome was Lin’s concordance correlation coefficient (CCC) between the height-based formula prediction [(height in cm/10) + 5] and the radiographically confirmed ETT insertion depth at the lip level. The mean predicted depth was 11.54 cm versus the mean observed X-ray depth of 11.63 cm, a difference of only −0.09 cm. The Pearson correlation was 0.717 (*p* < 0.001), reflecting reasonable precision. However, the CCC was 0.694 (95% CI: 0.605–0.766), which falls below the pre-specified threshold of 0.75, indicating poor concordance. The height-based formula, therefore, did not meet the primary outcome criterion for acceptable agreement with the radiographic standard.

All five formulas demonstrated poor concordance with the X-ray-confirmed insertion depth (CCC < 0.75 for all) (Table 1). Among the three classical formulas, the height-based formula performed best (CCC 0.694, 95% CI: 0.605–0.766), followed closely by the ETT size-based formula (CCC 0.680, 95% CI: 0.563–0.769), and then the weight-based formula (CCC 0.572, 95% CI: 0.487–0.650). The two Lee formulas were substantially worse, with the Lee height-based formula achieving a CCC of only 0.316 (95% CI: 0.252–0.376) and the Lee weight-based formula the lowest CCC of 0.256 (95% CI: 0.200–0.309). Despite similar Pearson r values between the height-based and Lee formulas (both 0.717), the large systematic offset of the Lee formulas sharply reduced their CCC.

### 3.6. Bland–Altman Analysis

**Figure 3:**
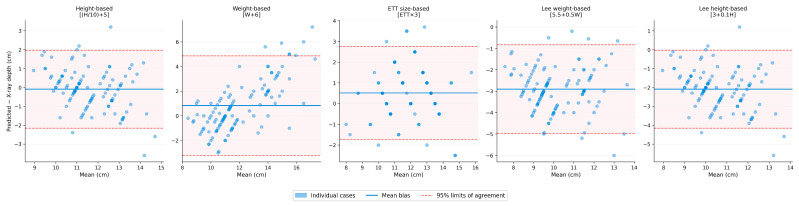
Bland–Altman plots comparing formula-predicted and X-ray-confirmed ETT insertion depth for all five formulas (n = 115). Each panel shows individual patient data points, the mean bias (solid blue line), and the 95% limits of agreement (dashed red lines). Bias values are annotated on the right of each panel. The three classical formulas (height-based, weight-based, ETT size-based) show scatter around zero, while both Lee formulas demonstrate consistent systematic underestimation, with their entire point clouds shifted below zero and limits of agreement that do not cross the zero line.

The height-based formula had the smallest systematic bias of all five formulas (−0.09 cm), indicating negligible average over- or under-prediction and relatively narrow limits of agreement (−2.16 to +1.98 cm). The ETT size-based formula had a modest positive bias (+0.51 cm) with a similarly narrow spread (−1.73 to +2.76 cm). The weight-based formula overestimated by a mean of +0.82 cm and had the widest limits of agreement (−3.21 to +4.86 cm), reflecting high inter-patient variability (Figure 3). Both Lee formulas showed large, consistent systematic underestimation: Lee weight-based (bias −2.90 cm, LoA −4.97 to −0.83 cm) and Lee height-based (bias −2.09 cm, LoA −4.16 to −0.02 cm). The Lee formula LoAs did not cross zero, confirming they never reliably approached the true insertion depth in this population (Figure 3).

### 3.7. Clinical Classification of Formula Predictions

The ETT size-based formula achieved the highest rate of ideal predictions (|error| ≤ 0.5 cm from X-ray depth) at 48.7%, followed by the height-based formula at 43.5%. Including the acceptable range (error > 0.5 to ≤1.0 cm), the combined ideal-or-acceptable rates were 73.9% for ETT size-based and 71.3% for height-based, closely matched, and the two highest of all formulas. The weight-based formula was ideal in only 26.1% and unacceptable in 54.8% of cases. Both Lee formulas failed markedly: the Lee weight-based formula was unacceptable in 96.5% of patients, and the Lee height-based formula in 86.1% (Table 2).

### 3.8. Direction of Predicted Malposition

The height-based formula showed a balanced error pattern: 29 cases (25.2%) were placed too deep, 36 (31.3%) too shallow, and 50 (43.5%) were correctly predicted. The weight-based formula predominantly overestimated (too deep: 56, 48.7%), and the ETT size-based formula also tended to overestimate (too deep: 47, 40.9%) but had the highest proportion of correct predictions (56, 48.7%). Both Lee formulas showed near-complete systematic underestimation: the Lee weight-based formula predicted too shallow in 113 of 115 cases (98.3%) and the Lee height-based formula in 106 of 115 (92.2%), with virtually no overestimation, confirming systematic miscalibration for this age group (Table 3).

### 3.9. Stratified Regression Analysis by Age Subgroup

**Figure 4:**
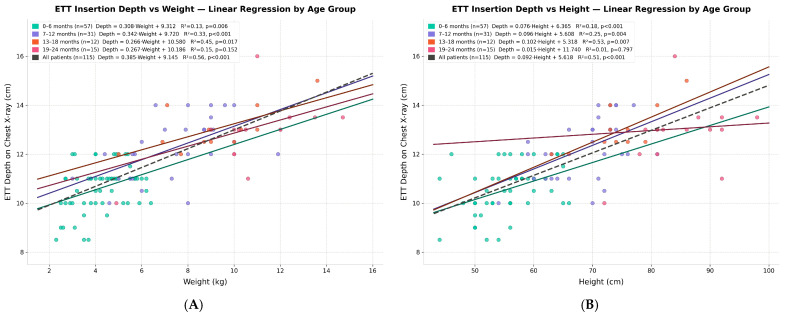
Scatter plots of radiographically confirmed ETT insertion depth (cm) against weight (kg) (**A**) and height (cm) (**B**) for all 115 patients, stratified by age groups. Colored circles and solid lines represent individual data points and regression lines for each age subgroup. The dashed black line represents the overall cohort regression line (all 115 patients combined).

To examine whether the relationship between anthropometric predictors and radiographically confirmed ETT insertion depth varied across the age spectrum, the cohort was stratified into four predefined subgroups: 0–6 months (n = 57), 7–12 months (n = 31), 13–18 months (n = 12), and 19–24 months (n = 15). Simple linear regression was performed for weight and height independently within each stratum and across the full cohort (Figure 4).

For weight-based regression across the whole cohort, the derived equation was Depth = 0.385 × Weight (kg) + 9.145 (R^2^ = 0.557, *p* < 0.001). Within subgroups, the slope and goodness-of-fit were highest in the 7–12 month stratum (Depth = 0.342 × Weight + 9.720; R^2^ = 0.325, *p* < 0.001) and the 13–18 month stratum (Depth = 0.266 × Weight + 10.580; R^2^ = 0.452, *p* = 0.017). In the youngest infants (0–6 months), weight accounted for only 13% of variance in insertion depth (R^2^ = 0.129, *p* = 0.006). In the 19–24 month group, the weight-based regression did not reach statistical significance (R^2^ = 0.151, *p* = 0.152), reflecting both the small sample size and increasing inter-patient variability at this age.

For height-based regression across the whole cohort, the derived equation was Depth = 0.092 × Height (cm) + 5.618 (R^2^ = 0.514, *p* < 0.001). Subgroup performance followed a similar pattern: goodness-of-fit was moderate in the 13–18 month stratum (R^2^ = 0.529, *p* = 0.007) and the 7–12 month stratum (R^2^ = 0.251, *p* = 0.004), but markedly weaker in infants aged 0–6 months (R^2^ = 0.183, *p* < 0.001). Notably, height lost all predictive value in the 19–24 month group, with a near-zero slope (0.015 cm per cm) and non-significant association (R^2^ = 0.005, *p* = 0.797), suggesting that height variation within this narrow age band does not translate into meaningful differences in ETT insertion depth.

These findings are summarized in Table 4 and illustrated in Figure 4. Across all subgroups, no single anthropometric predictor achieved an R^2^ exceeding 0.56, and the addition of age to a multivariable model combining age, weight, and height produced only a negligible improvement in explained variance (R^2^ = 0.569 for all patients combined), confirming that the ceiling of linear single- and multi-predictor modeling has been reached in this cohort. The progressive attenuation of predictor–depth associations with advancing age—and the complete failure of height-based regression in the 19–24 month stratum—underscores that pooled linear formulas impose artificial uniformity on a population in whom the anthropometric–airway relationship is both non-linear and age-dependent.

## 4. Discussion

This study evaluated five commonly used ETT depth prediction formulas in 115 infants and young children aged 1–24 months admitted to a tertiary PICU. The central finding is that none of the five formulas achieved acceptable concordance with radiographically confirmed insertion depth, with all CCC values falling below the pre-specified threshold of 0.75. Among the classical formulas, the height-based formula [(H/10) + 5] performed best—with the smallest bias (−0.09 cm), the narrowest limits of agreement, and the highest combined ideal-or-acceptable clinical classification rate (71.3%)—but its CCC of 0.694 still reflects insufficient reliability for individual patient use without radiographic confirmation.

The apparent paradox that the height-based formula achieved a poor CCC while correctly predicting depth within an acceptable range in nearly three-quarters of cases reflects the fact that these two metrics answer fundamentally different questions. The clinical classification rate measures how often a formula lands within a predefined distance of the X-ray depth and is insensitive to errors beyond that threshold. The CCC penalizes both systematic bias and inter-patient scatter simultaneously across the full distribution of paired values [24]. In a young infant where the entire tracheal length is only 4–5 cm, a prediction error of 2 cm represents displacement of nearly half the trachea, making the CCC the more clinically honest measure of individual-level reliability [25]. Put simply, the formula works reasonably well on average but cannot be trusted for any given patient.

The most directly comparable published study applied the same analytical framework in 30 children aged 29 days to 2 years from a cardiac PICU, reporting a CCC of 0.88 for the height-based formula with an ideal classification rate of 43.3%—virtually identical to ours (43.5%) [1]. Despite the higher CCC, that study still concluded that formula-based depth estimation cannot replace X-ray confirmation [1]. The difference in CCC between the two studies most plausibly reflects the greater homogeneity of that cardiac cohort, which produced less inter-patient scatter. Our larger, more heterogeneous PICU sample more faithfully represents the variability encountered in general clinical practice, and the consistency of the ~43% ideal classification rate across two independent cohorts suggests this may represent a ceiling inherent to linear formula-based prediction in this age group.

The height-based formula was originally derived from children aged 3–16 years, in whom the distance from the teeth to the mid-trachea was found to correlate strongly with height, yielding the equation height (cm)/10 + 5 [6]. This origin-mismatch is a likely contributor to its limited performance in the 1–24-month range, where body proportions and tracheal growth are non-linear—a point demonstrated empirically by Ebenebe et al., who showed that all published linear formulas induce considerable malposition rates in children precisely because they impose linearity on non-linear growth [9]. Nevertheless, the height-based formula’s balanced error pattern (with no systematic directional bias and roughly equal rates of over- and under-prediction) makes it the most clinically defensible bedside tool among those tested, with the additional practical advantage that height can be conveniently estimated using the Broselow tape [9].

Beyond validating existing published formulas, this study derived weight- and height-based linear regression equations directly from this cohort, representing the first such equations calibrated specifically to children under two years in a general PICU setting. Strikingly, weight emerged as the stronger predictor, explaining 55.7% of the variance in radiographic insertion depth (*p* < 0.001), compared with 51.4% for height (*p* < 0.001). These values compare favorably with published formulas, none of which were derived from this age group in a general PICU context. Notably, weight is universally available at the bedside, can be estimated reliably even in a critically ill infant, and does not require accurate length measurement, which is notoriously difficult in sedated or restrained patients. The weight-based formula derived here (Depth = 0.385 × Weight + 9.145) thus offers a more physiologically appropriate and practically accessible starting point for initial depth estimation in children under two years than any of the published formulas tested, and warrants prospective validation as a clinical decision-support tool.

The ETT size-based formula [ETT × 3] achieved a marginally higher ideal classification rate (48.7%) but showed a systematic tendency to overestimate depth (too deep in 40.9% of cases versus too shallow in only 10.4%), raising preferential concern for endobronchial intubation—arguably the more dangerous error in this population. This asymmetry aligns with findings from a prospective evaluation of intubated PICU patients, in which the ETT × 3 rule resulted in a significant proportion of malpositioning, partly because the stepwise increments imposed by discrete tube sizes produce a minimum depth-step of 15 mm—a particularly problematic increment in small infants [5,9]. The weight-based formula [W + 6] had the widest limits of agreement and the lowest ideal classification rate among the classical formulas (26.1%), consistent with the known instability of weight as a predictor of tracheal length in young infants, particularly those with chronic illness or oedema common in a PICU setting, and consistent with prior reports that weight-based linear formulas demonstrate acceptable accuracy only within a limited weight range [1,9].

Both Lee formulas performed dramatically worse than any of the classical formulas, producing near-universal systematic underestimation: the Lee weight-based formula [5.5 + 0.5W] predicted too shallow in 113 of 115 patients (98.3%), and the Lee height-based formula [3 + 0.1H] in 106 of 115 (92.2%), with unacceptable errors in 96.5% and 86.1% of cases, respectively. These formulas were derived from CT-measured anatomical tracheal lengths—the distance from the upper incisor to the mid-trachea on sagittal neck CT images—in 1111 children, not from clinical intubation data [7]. The severe underestimation observed here likely reflects a fundamental gap between the CT-derived anatomical measurement and the clinically confirmed lip-level insertion depth. The clinical implication is unambiguous: the Lee formula should not be applied in infants aged 1–24 months without prospective validation in this population, as its use would result in near-systematic under-insertion and a high risk of accidental extubation.

The 51.3% malposition rate at the time of the first post-intubation X-ray—before any formula-guided correction—underscores that the challenge is not merely one of formula selection but of the fundamental difficulty of accurate ETT placement in the shortest and most rapidly growing tracheas. A prospective PICU study of mixed-age patients reported a malposition rate of 23% [5], and a retrospective PICU cohort using cuffed tubes found an inappropriately deep tip position in 23.5% of cases, identifying small height, a history of abdominal disease, and oversized ETT as independent risk factors [26]. The higher malposition rate in our cohort likely reflects the greater clinical acuity and narrow age distribution of our population, and reinforces that post-intubation radiographic verification cannot be regarded as optional in this age group.

The findings of this study reinforce that no currently available bedside adjunct can substitute for radiographic depth verification in this age group. Point-of-care ultrasound (POCUS) has an established and growing role in confirming tracheal ETT placement, a systematic review of 33 studies in neonates, infants, and older children reported diagnostic accuracy of 90.6–100% for confirming successful tracheal intubation [13], prospective PICU validation demonstrated accuracy exceeding 97% against a radiographic standard [15], and the 2024 ESAIC/BJA joint guidelines endorse POCUS as a useful intubation adjunct [12], but POCUS answers only the binary question of tracheal versus esophageal placement. Its accuracy in quantifying ETT tip-to-carina distance varies widely (66.7–100%) [13], and, from a neonatologist’s perspective, reliable tip localization by ultrasound alone remains insufficient to determine safe carina clearance in clinical practice [16]. In the present cohort, where 40.0% of tubes were too deep and 11.3% too shallow at the first post-intubation X-ray, this distinction is clinically critical: a tube confirmed as tracheal by ultrasound may still be endobronchial. Looking further ahead, machine learning (ML) approaches represent the most promising direction beyond the ceiling demonstrated by linear formulas in this study. In pediatric patients under 7 years, ML models achieved optimal ETT depth prediction in up to 79.0% of cases, compared with only 44.4% for the height-based formula [19], and a dedicated pediatric ML study confirmed significant outperformance over all conventional formula comparators [20]. Deep learning applied to chest radiographs has additionally demonstrated sensitivity and specificity exceeding 90% for detecting critically malpositioned ETTs, with ETT-to-carina distance prediction within 1 cm in most cases [18]—a capability that could deliver near-real-time automated flagging of malposition without the delays of formal radiology reporting. The data generated in the present study, including the age-stratified predictor–depth relationships and the empirical ceiling of linear R^2^ at 0.557, provide both the motivation and the benchmarks for future ML model development in this population.

### Limitations

Several limitations should be acknowledged. The retrospective, single-center design at a tertiary PICU in Riyadh limits generalizability, and the anthropometric characteristics of the Saudi infant population may differ from those of the derivation populations. For all five formulas tested, none specifically targeted infants aged 1–24 months in a general PICU context. All tubes were uncuffed; findings and the cohort-derived regression equations may not apply to centers routinely using cuffed ETTs in this age group, as cuffed and uncuffed tubes differ structurally in ways that affect the relationship between lip-level insertion depth and ETT tip position [10,12,23]. The Lee formulas’ poor performance may partly reflect age-group mismatch rather than formula failure per se, and prospective evaluation within their intended age strata using clinically confirmed insertion depths, rather than the CT-derived anatomical distances from which they were derived, is warranted. The cohort-derived regression equations were developed and tested within the same dataset and require prospective external validation before clinical adoption; the residual unexplained variance (R^2^ ≤ 0.557) and the negligible gain from multivariable modeling (R^2^ = 0.569) confirm that the ceiling of linear prediction has been reached, motivating future non-linear and machine-learning approaches [9,19,20]. Finally, retrospective design precluded causal attribution of complications to ETT malposition; the 82.6% overall complication rate reflects general PICU morbidity in critically ill infants, with ETT position-attributable events (endobronchial intubation and pneumothorax) occurring in 13.9% of patients.

## 5. Conclusions

None of the five tested formulas achieved acceptable concordance with radiographically confirmed ETT insertion depth in infants and young children aged 1–24 months. Among the published formulas, the height-based formula remains the most balanced bedside tool and may reasonably guide initial tube placement, but its individual-level unreliability means that post-intubation chest radiographic verification cannot be replaced by formula-based prediction alone and must remain the standard of care. Both Lee formulas showed near-universal systematic underestimation and should not be applied in this population without prospective validation.

This study derived novel weight- and height-based regression equations directly from a general PICU cohort of children under two years of age—the first such equations calibrated specifically to this population. These cohort-derived formulas outperform all five published formulas tested, with weight emerging as the strongest single predictor of insertion depth. We therefore recommend the following weight-based formula as the most appropriate starting point for initial ETT depth estimation in children aged 1–24 months: Depth (cm) = 0.385 × Weight (kg) + 9.145. This formula is simple, requires only bedside weight (universally available even in critically ill infants), and warrants prospective external validation before routine clinical adoption. Future research should explore non-linear and machine-learning approaches, where the linear assumptions underlying all current formulas are least likely to hold.

## Figures and Tables

**Table 1:** Primary and Secondary Outcome—Lin’s CCC, Pearson r, MAE, and RMSE for All Five Formulas (n = 115).

Formula	Mean Predicted (cm)	Mean X-Ray (cm)	Pearson r	CCC	95% CI	MAE (cm)	RMSE (cm)
Height-based [(H/10)+5]	11.54	11.63	0.717	0.694	0.605–0.766	0.79	1.05
Weight-based [W+6]	12.45	11.63	0.746	0.572	0.487–0.650	1.64	2.21
ETT size-based [ETT×3]	12.14	11.63	0.719	0.680	0.563–0.769	0.95	1.25
Lee weight-based [5.5+0.5W]	8.73	11.63	0.746	0.256	0.200–0.309	2.90	3.09
Lee height-based [3+0.1H]	9.54	11.63	0.717	0.316	0.252–0.376	2.11	2.34

CCC = Lin’s concordance correlation coefficient; 95% CI = bootstrap 95% confidence interval (5000 resamples, paired); MAE = mean absolute error; RMSE = root mean square error. CCC thresholds: >0.90 good; 0.75–0.90 moderate; <0.75 poor. All comparisons are between the formula-predicted insertion depth and the X-ray-confirmed insertion depth at the lip/teeth level.

**Table 2:** Clinical Classification of Formula Predictions Relative to X-ray-Confirmed ETT Insertion Depth (n = 115).

Formula	Ideal	Acceptable	Unacceptable	Ideal + Acceptable
Height-based [(H/10)+5]	50 (43.5%)	32 (27.8%)	33 (28.7%)	82 (71.3%)
Weight-based [W+6]	30 (26.1%)	22 (19.1%)	63 (54.8%)	52 (45.2%)
ETT size-based [ETT×3]	56 (48.7%)	29 (25.2%)	30 (26.1%)	85 (73.9%)
Lee weight-based [5.5+0.5W]	2 (1.7%)	2 (1.7%)	111 (96.5%)	4 (3.5%)
Lee height-based [3+0.1H]	8 (7.0%)	8 (7.0%)	99 (86.1%)	16 (13.9%)

Ideal: absolute difference between predicted and X-ray depth ≤ 0.5 cm; Acceptable: >0.5 to ≤1.0 cm; Unacceptable: >1.0 cm. Values are n (%).

**Table 3:** Direction of Predicted Malposition Relative to X-ray-Confirmed ETT Insertion Depth (n = 115).

Formula	Too Deep	Too Shallow	Correct
Height-based [(H/10)+5]	29 (25.2%)	36 (31.3%)	50 (43.5%)
Weight-based [W+6]	56 (48.7%)	29 (25.2%)	30 (26.1%)
ETT size-based [ETT×3]	47 (40.9%)	12 (10.4%)	56 (48.7%)
Lee weight-based [5.5+0.5W]	0 (0.0%)	113 (98.3%)	2 (1.7%)
Lee height-based [3+0.1H]	1 (0.9%)	106 (92.2%)	8 (7.0%)

Too deep: formula predicts an insertion depth > 0.5 cm deeper than X-ray reference (tube would sit closer to or past the carina). Too shallow: formula predicts an insertion depth > 0.5 cm shallower than X-ray reference (tube would sit higher in the airway). Values are n (%).

**Table 4:** Stratified linear regression of radiographic ETT insertion depth on weight and height, by age subgroup.

Age Group	n	Predictor	Regression Formula	R^2^	*p*-Value
0–6 months	57	Weight (kg)	Depth = 0.308 × Weight + 9.312	0.129	0.006 *
		Height (cm)	Depth = 0.076 × Height + 6.365	0.182	<0.001 *
7–12 months	31	Weight (kg)	Depth = 0.342 × Weight + 9.720	0.325	<0.001 *
		Height (cm)	Depth = 0.096 × Height + 5.608	0.251	0.004 *
13–18 months	12	Weight (kg)	Depth = 0.266 × Weight + 10.580	0.452	0.017 *
		Height (cm)	Depth = 0.102 × Height + 5.318	0.529	0.007 *
19–24 months	15	Weight (kg)	Depth = 0.267 × Weight + 10.186	0.151	0.152
		Height (cm)	Depth = 0.015 × Height + 11.741	0.050	0.797
All patients	115	Weight (kg)	Depth = 0.385 × Weight + 9.145	0.557	<0.001 *
		Height (cm)	Depth = 0.092 × Height + 5.618	0.514	<0.001 *

Depth = radiographically confirmed ETT insertion depth (cm). Predictor variables: Weight (kg) and Height (cm). R^2^ = coefficient of determination, expressed as the percentage of variance in ETT insertion depth explained by the predictor variable alone. *—statistically significant.

## Data Availability

Data are available from the corresponding author upon reasonable request.

## Abbreviations

AP	Anteroposterior
CCC	Concordance Correlation Coefficient
CI	Confidence Interval
ETT	Endotracheal Tube
ICC	Intraclass Correlation Coefficient
IQR	Interquartile Range
LoA	Limits of Agreement
MAE	Mean Absolute Error
PA	Posteroanterior
PACS	Picture Archiving and Communication System
PICU	Pediatric Intensive Care Unit
RMSE	Root Mean Square Error
SD	Standard Deviation
T1–T2	First to Second Thoracic Vertebrae

## References

[1] Santos D.L.S., Andrade P.D.O., Gomes E.L.F.D. (2020). Does the endotracheal tube insertion depth predicted by formulas in children have a good concordance with the ideal position observed by X-ray?. Rev. Bras. Ter. Intensiva.

[2] Harris E.A., Arheart K.L., Penning D.H. (2008). Endotracheal tube malposition within the pediatric population: A common event despite clinical evidence of correct placement. Can. J. Anaesth..

[3] Weiss M., Knirsch W., Kretschmar O., Dullenkopf A., Tomaske M., Balmer C., Stutz K., Gerber A.C., Berger F. (2006). Tracheal tube-tip displacement in children during head-neck movement—A radiological assessment. Br. J. Anaesth..

[4] Trout S., Aaron J., Zapata-Sirvent R.L., Hansbrough J.F. (1994). Influence of head and neck position on endotracheal tube tip position on chest x-ray examination: A potential problem in the infant undergoing intubation. J. Burn. Care Rehabil..

[5] Phipps L.M., Thomas N.J., Gilmore R.K., Raymond J.A., Bittner T.R., Orr R.A., Robertson C.L. (2005). Prospective assessment of guidelines for determining appropriate depth of endotracheal tube placement in children. Pediatr. Crit. Care Med..

[6] Morgan G.A.R., Steward D.J. (1982). Linear airway dimensions in children: Including those from cleft palate. Can. Anaesth. Soc. J..

[7] Lee S.U., Jung J.Y., Kim D.K., Kwak Y.H., Kwon H., Cho J.H., Park J.W., Choi Y.J. (2018). New decision formulas for predicting endotracheal tube depth in children: Analysis of neck CT images. Emerg. Med. J..

[8] Luten R.C., Wears R.L., Broselow J., Zaritsky A., Barnett T.M., Lee T., Bailey A., Vally R., Brown R., Rosenthal B. (1992). Length-based endotracheal tube and emergency equipment in pediatrics. Ann. Emerg. Med..

[9] Ebenebe C.U., Schriever K., Apostolidou S., Wolf M., Herrmann J., Singer D., Deindl P. (2023). Recommendations for endotracheal tube insertion depths in children. Emerg. Med. J..

[10] Weiss M., Gerber A.C., Dullenkopf A. (2005). Appropriate placement of intubation depth marks in a new cuffed paediatric tracheal tube. Br. J. Anaesth..

[11] Gottlieb M., Holladay D., Peksa G.D. (2018). Ultrasonography for the confirmation of endotracheal tube intubation: A systematic review and meta-analysis. Ann. Emerg. Med..

[12] Disma N., Asai T., Cools E., Cronin A., Engelhardt T., Fiadjoe J., Fuchs A., Garcia-Marcinkiewicz A., Habre W., Heath C. (2024). Airway management in neonates and infants: European Society of Anaesthesiology and Intensive Care and British Journal of Anaesthesia joint guidelines. Br. J. Anaesth..

[13] Liu Y., Ma W., Liu J. (2023). Applications of airway ultrasound for endotracheal intubation in pediatric patients: A systematic review. J. Clin. Med..

[14] Congedi S., Savio F., Auciello M., Salvadori S., Nardo D., Bonadies L. (2022). Sonographic evaluation of the endotracheal tube position in the neonatal population: A comprehensive review and meta-analysis. Front. Pediatr..

[15] Chandnani H.K., Maxson I.N., Mittal D.K., Dehom S., Moretti A., Dinh V.A., Lopez M., Ejike J.C. (2021). Endotracheal tube placement confirmation with bedside ultrasonography in the pediatric intensive care unit: A validation study. J. Pediatr. Intensive Care..

[16] Sahin O., Tasar S., Colak D., Yavanoglu Atay F., Guran O., Mungan Akin I. (2024). Point-of-care ultrasound for the tip of the endotracheal tube: A neonatologist perspective. Am. J. Perinatol..

[17] Kim H., Yoon H.K., Lee H., Jung C.W., Lee H.C. (2023). Predicting optimal endotracheal tube size and depth in pediatric patients using demographic data and machine learning techniques. Korean J. Anesthesiol..

[18] Lakhani P., Flanders A., Gorniak R. (2021). Endotracheal tube position assessment on chest radiographs using deep learning. Radiol. Artif. Intell..

[19] Shim J.G., Ryu K.H., Lee S.H., Cho E.A., Lee S., Ahn J.H. (2021). Machine learning model for predicting the optimal depth of tracheal tube insertion in pediatric patients: A retrospective cohort study. PLoS ONE.

[20] Shim J.G., Lee E.K., Oh E.J., Cho E.A., Park J., Lee J.H., Ahn J.H. (2023). Predicting the risk of inappropriate depth of endotracheal intubation in pediatric patients using machine learning approaches. Sci. Rep..

[21] Lau N., Playfor S.D., Rashid A., Dhanarass M. (2006). New formulae for predicting tracheal tube length. Paediatr. Anaesth..

[22] Song I.K., Kim S.H., Ryu J., Lee E., Oh H.M., Kim E.H., Lee J.H., Kim H.S., Kim J.T. (2016). Prediction of the midtracheal level based on external anatomical landmarks: Implication of the optimal insertion depth of endotracheal tubes in pediatric patients. Paediatr. Anaesth..

[23] Khine H.H., Corddry D.H., Kettrick R.G., Martin T.M., McCloskey J.J., Rose J.B., Theroux M.C., Zagnoev M. (1997). Comparison of cuffed and uncuffed endotracheal tubes in young children during general anesthesia. Anesthesiology.

[24] Lin L.I. (1989). A concordance correlation coefficient to evaluate reproducibility. Biometrics.

[25] Bland J.M., Altman D.G. (1986). Statistical methods for assessing agreement between two methods of clinical measurement. Lancet.

[26] Matsuoka W., Ide K., Matsudo T., Kobayashi T., Nishimura N., Nakagawa S. (2019). The occurrence and risk factors of inappropriately deep tip position of Microcuff pediatric endotracheal tube during PICU stay: A retrospective cohort pilot study. Pediatr. Crit. Care Med..

